# Predictors of Health-Care Utilization Among Children 6–59 Months of Age in Zambézia Province, Mozambique

**DOI:** 10.4269/ajtmh.16-0233

**Published:** 2017-02-08

**Authors:** Mary Bayham, Meridith Blevins, Melanie Lopez, Omo Olupona, Lazaro González-Calvo, Elisée Ndatimana, Ann F. Green, Troy D. Moon

**Affiliations:** 1 Vanderbilt Institute for Global Health, Nashville, Tennessee.; 2 Friends in Global Health, Maputo, Mozambique.; 3 Department of Biostatistics, Vanderbilt University, Nashville, Tennessee.; 4 World Vision United States, Federal Way, Washington.; 5 World Vision International, Maputo, Mozambique.; 6 Division of Infectious Diseases, Department of Pediatrics, Vanderbilt University, Nashville, Tennessee.

## Abstract

Globally, approximately 5.9 million children under 5 years of age died in 2015, a reduction of over 50% since 1990. Millennium Development Goal 4 established the goal of reducing child mortality by two-thirds by 2015. Multiple countries have surpassed this goal; however, regional and within-country inequities exist. We sought to study determinants of health-care utilization among children 6–59 months of age with fever, diarrhea, and respiratory symptoms in Zambézia Province, Mozambique. We conducted a population-based cross-sectional survey of female heads of household between April and May 2014. Mobile teams conducted interviews in 262 enumeration areas, with three distinct districts being oversampled for improved precision. Descriptive statistics and logistic regression using Stata 13.1 and R 3.2.2 were used to examine factors associated with health-care utilization. A total of 2,317 children were evaluated in this study. Mothers' median age was 26 years, whereas child median age was 24 months. The proportion of children reporting fever, diarrhea, or respiratory illness in the prior 30 days was 44%, 22%, and 22%, respectively. Health-care utilization varied with 65% seeking health care for fever, compared with 57% for diarrhea and 25% for respiratory illness. In multivariable logistic regression, the characteristics most associated with health-care utilization across illnesses were delivery of last child at a facility, higher maternal education, and household ownership of a radio. The decision or ability to use health care is a multifaceted behavior swayed by societal norms, values, socioeconomics, and perceived need. Recognizing the predictors of a particular population may offer useful information to increase uptake in health-care services.

## Background

Globally, an estimated 5.9 million children under 5 years of age died in 2015, a reduction of over 50% since 1990.^1^,^2^ The Millennium Development Goals (MDGs), committed to by world leaders at the United Nations Summit in 2000, have produced the most successful health and development movement in history.^3^ MDG 4 established the goal of reducing child mortality rates by two-thirds between 1990 and 2015, and as of December 2015, several countries in Africa appear to have surpassed this goal. Despite these achievements, Africa still has the highest proportion of under-five mortality and accounts for almost half of all child deaths in the world.^1^,^4^ Additionally, within countries, regions have fallen short, indicating that continued effort is essential in this area.^4^

**Figure 1.**
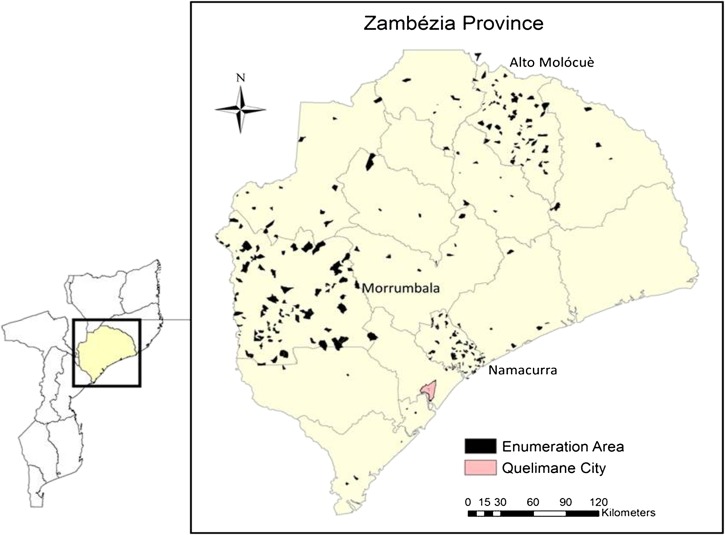
Map of Mozambique highlighting Zambézia Province and Maputo Province, with enumeration areas enlarged. Charlotte Buehler; March 3, 2016; Vanderbilt Institute for Global Health; Projection: WGS 1984 Web Mercator Auxiliary Sphere. Permission has been granted by the copy-right holder for publication of this figure in an open access journal.

In 2015, Mozambique ranked 180 of 188 countries on the United Nations Development Program Human Development Index,^5^ having the 23rd highest national under-five mortality rate at 79 per 1,000 live births.^1^,^6^ This rate has been falling over time with a 2005 national under-five mortality estimated at 138 per 1,000 live births compared with 219 per 1,000 live births in 1997.^6^–^9^ Although Mozambique appears to have surpassed its 2015 national under-five mortality target for MDG 4, substantial regional differences persist.^10^ Economic activity, infrastructure, and basic services are highly concentrated around the capital Maputo, which is located in the southernmost part of the country. In 2011, Maputo Province had the lowest under-five mortality (96 per 1,000 live births) compared with Zambézia Province (142 per 1,000 live births), which is among the highest risk provinces in Mozambique for under-five mortality (Figure 1

).^11^,^12^ Additionally, Zambézia Province has among the lowest access to safe drinking water and sanitation among children (26% and 73%, respectively), as well as some of the lowest performance indicators for health outcomes in Mozambique.^11^ As a result, Zambézia has been labeled a priority province for development efforts.^13^

The majority of childhood deaths in Mozambique are caused by infectious, preventable, and treatable conditions including malaria, acute respiratory infections, diarrhea, and acquired immunodeficiency syndrome.^1^,^2^,^11^,^14^ Together, these conditions account for approximately 50% of all under-five mortality in Mozambique.^11^ The preventable, treatable nature of the leading causes of child mortality indicates that increased utilization of health-care facilities could reduce the burden of child mortality attributed to these illnesses. Since its independence in 1975, the Government of Mozambique has attempted to increase access to health services by expanding primary care services and removing fees for children under 5 years of age.^11^,^14^ The dominant assumption in public health is that availing these services to individuals will result in increased utilization.^15^,^16^ However, uptake of health services for children under 5 years of age is more complex.

The determinants of health-care utilization range from socioeconomic to system-level factors including religious and cultural beliefs, age, education, sex, household income, travel time, and others.^15^–^19^ Understanding behaviors of health-care utilization for fever, diarrhea, and respiratory illness in Mozambique are even more important because these symptoms are often considered trivial to the caretaker, or not requiring immediate attention, despite their high association with under-five morbidity and mortality. These have been studied in various locations worldwide,^10^,^11^,^13^,^14^,^18^,^20^–^23^ but few studies include Mozambique.^9^,^24^ As we move past the MDGs and begin preparing to meet the new Sustainable Development Goals of ending under-five mortality by 2030,^25^ a more in-depth understanding of the social, biologic, and epidemiologic factors contributing to continued under-five mortality, will be required. Although the MDGs brought incredible advances globally, there is growing recognition that inequities persist and that the existing focus on national averages may actually have exacerbated regional and within-country inequalities, particularly for child health.^21^–^23^,^26^–^28^ Addressing these inequalities with a focus that targets interventions at a more regional and local level may help us reduce the disparities in childhood mortality that remain in low- and middle-income countries today.^29^–^31^ To this end, we sought to study the determinants of health-care utilization among children 6–59 months of age with fever, diarrhea, and respiratory symptoms in Zambézia Province, so as to inform future planning, policy, and interventions in reducing under-five morbidity and mortality in Mozambique.

## Methods

We used cross-sectional data from a household survey that was conducted as part of the Ogumaniha project (meaning “united for a common purpose” in the local Echuabo language), funded under the U.S. Agency for International Development (USAID) Strengthening Communities through Integrated Programming award. Ogumaniha's goal was to reduce poverty and improve the health of people living in Zambézia Province through cohesive community-based programming.^32^ A strong monitoring system and project evaluation based on performance indicators were agreed upon with USAID and the provincial government and were central to Ogumaniha's design. This study is an extension of the monitoring and evaluation component of the Ogumaniha project and analyzes cross-sectional survey data collected at the project's conclusion (April and May 2014). The survey tool, developed by an interdisciplinary team of researchers, is a 500-item questionnaire covering eight dimensions and includes many questions borrowed from previous national surveys in Mozambique such as the Demographic Health Survey and Multiple Indicator Cluster Survey. The survey was designed to gather information from the female head of household on topics such as household demographics; economic status; health knowledge, attitudes, and practices; access to health services and products; access to improved water and sanitation; nutritional intake; and others. The female head of household was selected because, in Zambézia Province, she is thought to be the person most familiar with the health and caretaking of all household members (e.g., nutrition, water and sanitation, health events, and health-care access). There was a potential for bias in the case of polygamous families because the principal or eldest wife was selected, whereas the younger wives and their children may have been even more disadvantaged. A child health and immunization module collected child-specific information on up to two children 6–59 months of age per household. Mobile survey teams conducted interviews in 262 enumeration areas (EA). EA were selected in two-stage cluster sampling using the national census results as a sampling frame; first, we stratified by urban/rural grouping, and then sampled with probability proportional to size. Three districts (Alto Molócuè, Namacurra, and Morrumbala) were oversampled to improve accuracy for the primary project evaluation at reduced cost. A smaller, less dense sample of the remaining 14 districts was collected to provide survey-weighted estimates representative of the entire province. Further details about the sampling methodology, electronic data collection using mobile phones, Open Data Kit, and management protocols have been published elsewhere.^32^

In an effort to identify independent predictors of health-care utilization for children 6–59 months of age, we focused on the association between utilization and maternal education, household income, decision-making authority of the female head of household, and distance (in minutes) to a health facility. Covariates were selected a priori based on extensive literature review, and include child's age and sex, respondent's age, marital status, whether the respondent understands Portuguese, household size, mode of transport to a health-care facility, rural or urban location, ownership of a radio, and whether the respondent had delivered her last child in a health facility.

Descriptive statistics were calculated for continuous variables as weighted estimates of median (interquartile range [IQR]) and for categorical variables as weighted percentages, with each observation being weighted by the inverse of the household or child sampling probability. Outcomes of interest included health facility utilization after three common childhood illnesses: fever, diarrhea, and respiratory illness (symptoms included cough, difficulty breathing, or fast/shallow breathing). No definition for fever or diarrhea was provided by the study interviewer in order for the participant to respond based on their understanding of these conditions. Multivariable logistic regression models were used with robust covariance to account for clustering of children within households and EA. In each model, we disaggregated children with illness reported in the past 30 days and modeled the probability of health facility utilization. The significance level for all testing was two-sided and set at 0.05. If there was evidence of nonlinearity (*P* < 0.10) of continuous covariates with the log-odds of health facility utilization, then that variable was modeled using a restricted cubic spline. Multiple imputation was used to account for missing survey responses in covariates. We used the Hmisc package in R which used predictive mean matching to take random draws from imputation models; 10 imputation data sets were used in the analysis.^33^ Data analysis was conducted in Stata 13.1 and R version 3.2.2.^34^,^35^

Household participation in the survey was voluntary and without incentives. Written informed consent was obtained from the female head of household before the interview and child measurements were conducted. The study was approved by the Inter-institutional Bioethics Committee for Health of Zambézia Province (CIBS-Zambézia), Mozambique, and the Vanderbilt University Institutional Review Board.

## Results

A total of 2,317 children 6–59 months of age were measured in this study (Table 1). The provincial estimates of male and female children were 50% each. Maternal age was a median 26 years (IQR = 22–33), whereas child age was a median 24 months (IQR = 12–36). Most women (69%) were married or in a common law relationship. Province-wide, women had a median of 3 (IQR = 0–5) years of education, but overall education level was highest in Alto Molócuè at 4 years (IQR = 2–6). Eighty-three percent of female heads of household had a monthly income of less than 1,000 meticais (MZN) (1 USD = ∼31 MZN in April 2014). Seventy-eight percent of all respondents resided in rural areas, with rural residence in the three oversampled districts (Alto Molócuè, Morrumbala, and Namacurra) being 88%, 96%, and 100%, respectively.

Province-wide, fever, diarrhea, or respiratory illness in the 30 days before survey implementation, were 44%, 22%, and 22%, respectively (Table 2). Health-care utilization for these three conditions varied broadly with 65% of children being taken to a health facility for fever, compared with 57% for diarrhea and only 25% for respiratory illness.

When analyzed by our three oversampled districts, regional differences existed. The prevalence of children with fever in the prior 30 days was relatively similar across the three districts (39–48%), with similar proportions (65–67%) reporting visiting a health facility for the fever. However, the prevalence of diarrhea in the prior 30 days was higher in Alto Molócuè (31%), compared with Morrumbala (19%) and Namacurra (11%), though again similar proportions (51–56%) reported seeking care at a health facility. Finally, the prevalence of respiratory illness (cough, fast/shallow breath, or difficulty breathing) in the prior 30 days was again highest in Alto Molócuè (30%) compared with Morrumbala (24%) and Namacurra (13%). Yet in this instance, the proportion of children taken for care at a health facility varied with 40% in Alto Molócuè, 29% in Morrumbala, and only 13% in Namacurra.

**Figure 2.**
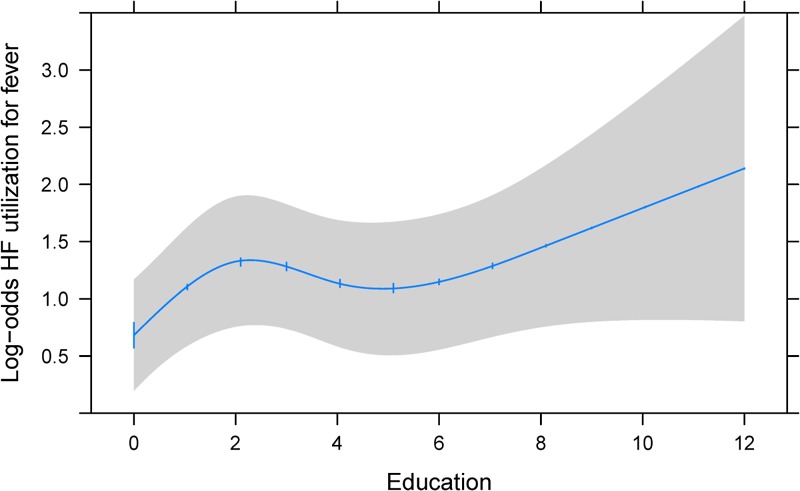
Adjusted association between maternal education and log-odds of health facility utilization for fever. Restricted cubic spline of association between maternal education (years) and the log-odds of health-care utilization for fever among children 6–59 months of age. The 95% confidence interval is represented by the gray area. Adjustment values of covariates include the median value or most prevalent category.

**Figure 3.**
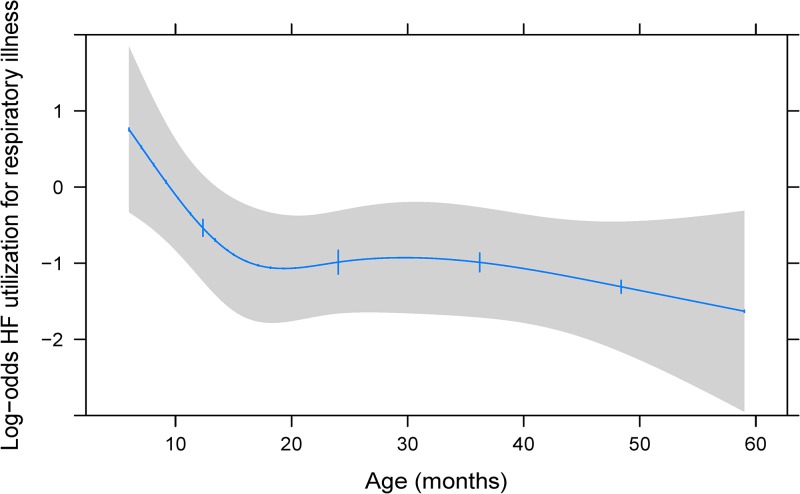
Adjusted association between child age (months) and log-odds of health facility utilization for respiratory illness. Restricted cubic spline of association between child age (months) and the log-odds of health-care utilization for respiratory illness among children 6–59 months of age. The 95% confidence interval is represented by the gray area. Adjustment values of covariates include the median value or most prevalent category.

In multivariable logistic regression models, the characteristics most associated with health-care utilization across all illnesses (fever, diarrhea, and respiratory illness) were delivery of last child at a health-care facility, higher maternal education, and household ownership of a radio (Table 3). Notably, if the respondent had delivered her last child at a health facility, she had a roughly 2–4 times higher odds of seeking care for her child under question (not necessarily the last child delivered) at a health facility for fever, diarrhea, or respiratory illness (diarrhea: odds ratio [OR] = 1.93; 95% confidence interval [CI] = 1.10, 3.37; *P* = 0.021; fever: OR = 2.69; 95% CI = 1.64, 4.40; *P* < 0.001; and respiratory illness: OR = 4.43; 95% CI = 2.91, 9.01; *P* < 0.001). Higher maternal education (5 years versus 0 years) was associated with a roughly 1.5 times higher odds of seeking care at a health facility for all three illness categories, though the observed association fell short of statistical significance for respiratory illness. Additionally, these data revealed a nonlinear association between maternal education and seeking care for fever, with the greatest increase in odds between 0 and 2 years of education, then increasing again for each year above 6 years (Figure 2

). Households with a radio had a roughly 1.5 times higher odds of seeking care at a health facility for all three illness categories (fever: OR = 1.42; 95% CI = 0.98, 2.05; *P* = 0.060; diarrhea: OR = 1.44; 95% CI = 0.99, 2.10; *P* = 0.058; and respiratory illness: OR = 1.78; 95% CI = 1.17, 2.72; *P* = 0.007). As travel time to a health facility increased (per 1 hour), respondents had a 19% lower odds of reported health facility utilization for diarrhea (OR = 0.81; 95% CI = 0.69, 0.94; *P* = 0.006) compared with an 18% and 36% lower odds for fever and respiratory illness, respectively. Respondents who reported living below the poverty line (< 1,000 MZN) had a 50% lower odds of taking their child to a health facility for diarrhea (OR = 0.50; 95% CI = 0.30–0.85; *P* = 0.009) compared with a 14% and 25% lower odds for fever and respiratory illness. There was little evidence of association between child age (12 versus 6 months) and a mother's decision to seek care at a health facility for either fever or diarrhea. However, mothers had a 71% lower odds of seeking health care for older children with respiratory illness compared with younger children (OR = 0.29; 95% CI = 0.12, 0.71; *P* < 0.001) (Figure 3

).

## Discussion

Our survey was implemented (April and May 2014) at the end of rainy season (typically December to April) when one would expect a seasonal elevation in childhood illnesses such as malaria, diarrhea, and respiratory illnesses.^36^–^38^ Overall, province-wide self-reported prevalence of these three conditions in children 6–59 months of age, in the 30 days before being questioned, was between 20% and 40% each. Caregivers sought care at a health facility in roughly 2/3 of fever cases, 1/2 of diarrhea cases, and only a 1/4 of respiratory illnesses. The decision or ability to use health services is a multifaceted behavior swayed by societal norms, values, socioeconomic factors, and perceived need.^14^,^18^,^19^,^39^–^42^ Recognizing the predictors of health-care utilization in a particular population may offer useful information to increase their uptake in services.

In our population, the factor most associated with health facility utilization across the three illnesses was delivery of last child at a health-care facility. Household ownership of a radio was significantly associated with health-care utilization, and was trending toward significance with diarrhea (*P* = 0.058) and fever (*P* = 0.06). Maternal education was further associated with health facility utilization for fever and diarrhea, whereas child age was associated with health facility utilization for respiratory illness. Multivariable analysis revealed that respondents who reported delivering their last child in a health facility had more than twice the odds of taking their child to a health facility. This finding aligns with our expectation that mothers who deliver in this setting will be more likely to use health facilities for their children. Future interventions for preventing unnecessary childhood deaths should focus on educating pregnant women about the advantages and safety of delivering at a health facility.

This study found that household ownership of a radio was associated with increased health-care utilization across all models. This is most likely due to that fact that in Mozambique, health campaigns are often conducted through mass media, with radio communication being one of the easiest and most frequent sources of health-care information dissemination, especially in the rural areas. Wealth and asset possession are widely recognized as predictors of health-care utilization.^13^,^19^,^40^–^43^ Our questionnaire asked the respondent to select the range of monthly household income that applies. We found that living below the poverty line (< 1,000 MZN, equivalent to approximately 1 USD per day in April 2014) was correlated with reduced utilization of biomedical health services for diarrhea. The absence of statistical significance across all models could be due to the fact that almost one quarter of respondents did not know their household income. Perhaps other measures of poverty, such as the multidimensional poverty index, might be more suitable.^13^ Ownership of electronics, such as television and radio, have been used as a proxy for wealth in other studies.^40^,^41^,^44^–^46^

Maternal education, specifically the first 2 years of schooling, increased the likelihood by approximately 50% that a mother would seek health care for her child with fever and diarrhea at a health facility. This result is consistent with the literature in which higher maternal education was found to be a significant predictor of health-care utilization.^19^,^40^,^47^–^49^ However, as years of education increased, the effect was less dramatic. Portuguese is the official language in Mozambique and the language most frequently used by physicians in the health-care facilities. For someone to speak Portuguese in rural Mozambique, it typically would require that they attend school for some period of time to have sufficient exposure enough to the language. As such, it can be postulated that mothers having at least 2 years of schooling could have learned enough Portuguese that they feel comfortable enough to enter into the health facility.

As a child grows older, the odds of being taken to a health facility for a respiratory illness decreased dramatically. This finding was not consistent for fever and diarrhea and could represent a limitation in our questionnaire items for respiratory illness. We defined respiratory illness as the child being reported to have either of the following symptoms; “cough” and/or “difficult or shallow breathing.” As a result, nonserious causes of cough such as allergies or simple viral respiratory infections not requiring medical attention could have overestimated the prevalence of “true” respiratory illness in our population. Also, it is possible that maternal knowledge of signs and symptoms of respiratory illness which may require medical attention are not clearly understood.

Despite mothers and/or caregivers knowing their child should be taken to a health facility, the issue is often one of access. The Mozambican Ministry of Health estimates that 60% of the population lives 20 miles or farther from any health facility.^14^,^50^ The necessity to travel long distances to a health-care facility is a barrier in health-care utilization, and is associated with increased child mortality.^51^ Longer travel time to a health facility was associated with decreased utilization in all models, but was statistically significant only in the diarrhea model. A potential limitation of our survey result is that the question regarding travel time to a health facility may have been misinterpreted, because the average travel time was lower than expected (median 4 minutes). The majority of Zambézia Province is rural, and 58.2% of its population is multidimensionally poor.^13^ We thought it pertinent to assess differences in urban and rural residence, as determinants of health, such as education and socioeconomic status, differ between the two.^7^,^9^,^10^,^18^,^24^,^29^,^42^,^52^,^53^

Strengths of this study include a large two-stage cluster survey sample of provincial data as opposed to countrywide data. Evidence of nonlinearity was further explored with restricted cubic splines in models that were developed a priori. Further research is needed to reproduce our findings in more settings to determine predictors in other diseases and populations. Specifically, researching predictors of antenatal care would be prudent because of the strong association it has with child health-care utilization. Moreover, incorporating both household surveys and health facility records would strengthen future studies of utilization. Understanding this behavior would lead to more effective interventions in preventing childhood death from treatable illness and improve the health and livelihood of the population.

## Figures and Tables

**Table 1:** Descriptive statistics of children 6–59 months of age, 2014

	Alto Molócuè (*N* = 697)	Morrumbala (*N* = 484)	Namacurra (*N* = 348)	All province (*N* = 2,317)
Child age (in months)	24 (12–36)	24 (12–26)	24 (12–36)	24 (12–36)
Mother's age (years) (18% missing)	27 (22–33)	29 (23–38)	27 (21–32)	26 (22–33)
Sex of child				
Male	49%	42%	49%	50%
Female	51%	58%	51%	50%
Understands Portuguese	62%	18%	43%	47%
Years of education (continuous)	4 (2–6)	1 (0–2)	1 (0–5)	3 (0–5)
Education category (respondent)				
0–5 years	73.5%	92.7%	76.8%	78.0%
6–10 years	25.4%	7.0%	21.4%	18.2%
> 10 years	1.1%	0.3%	1.7%	3.8%
Marital status				
Divorced/separated	2%	0.4%	6%	2%
Married/common law	89%	73%	57%	69%
Single	7%	25%	33%	27%
Widowed	2%	1%	5%	2%
Household size	5 (4–6)	5 (4–6)	4 (4–5)	5 (4–6)
Time to health facility (in minutes) (26% missing)	4 (2–90)	2 (1–4)	3 (3–90)	4 (2–20)
Urban/rural				
Rural	88%	96%	100%	78%
Urban	12%	4%	0%	22%
Decisions about seeking health care for child (*N* = 32, 1% missing)				
Men	15%	16%	9%	13%
Women	18%	12%	26%	30%
Both	68%	72%	66%	55%
Mode of transport to health facility (3% missing)				
Bicycle	21%	48%	10%	17%
Car	5%	1%	0%	1%
Motorcycle	4%	3%	2%	2%
On foot	71%	48%	88%	80%
Family income (24% missing)				
< 1,000 MZN per month	63%	74%	92%	83%
1,000 + MZN per month	37%	26%	8%	17%
Household has radio (*N* = 18 missing)	51%	52%	22%	35%
Respondent understands Portuguese	62%	18%	42%	47%

Continuous variables are reported as weighted estimates of median (interquartile range), with each observation being weighted by the inverse of the household sampling probability. Categorical variables are reported as weighted percentages, with each observation being weighted by the inverse of the household sampling probability.

**Table 2:** Health-care utilization in Zambézia Province, Mozambique, April–May 2014

	Alto Molócuè (*N* = 697)	Morrumbala (*N* = 484)	Namacurra (*N* = 348)	All province (*N* = 2,317)
Child had fever in last 30 days	46% (42, 50)	39% (31, 47)	48% (42, 53)	44% (38, 50)
Among those with fever, sought treatment at health facility	65% (56, 74)	67% (59, 75)	67% (57, 76)	64% (58, 71)
Child had diarrhea in last 30 days	31% (25, 36)	19% (15, 23)	11% (7, 15)	22% (19, 26)
Among those with diarrhea, sought treatment at health facility	56% (45, 67)	51% (36, 66)	56% (40, 73)	57% (49, 66)
Child has had respiratory illness last 30 days	30% (25, 36)	24% (19, 29)	13% (10, 16)	22% (18, 26)
Among those with respiratory illness, sought treatment at health facility	40% (33, 47)	29% (18, 39)	30% (19, 41)	25% (18, 32)

Categorical variables are reported as weighted percentages, with each observation being weighted by the inverse of the household sampling probability. The 95% confidence intervals include precision estimates that incorporate the effects of stratification and clustering.

**Table 3:** Health facility utilization among children under 5 years of age with fever, diarrhea, and respiratory illness: odds ratios from bivariate and multivariable logistic regression models

	Fever				Diarrhea				Respiratory illness			
	Unadjusted odds ratio (95% CI)	*P* value	Adjusted odds ratio (95% CI)	*P* value	Unadjusted odds ratio (95% CI)	*P* value	Adjusted odds ratio (95% CI)	*P* value	Unadjusted odds ratio (95% CI)	*P* value	Adjusted odds ratio (95% CI)	*P* value
Age (12 vs. 6 months)	1.39 (0.86, 2.26)	0.44	1.02 (0.96, 1.09)	0.51	1.52 (0.85, 2.72)	0.37	1.09 (0.99, 1.21)	0.085	0.41 (0.22, 0.77)	0.002	0.29 (0.12, 0.71)	< 0.001
Male vs. female child	1.00 (0.73, 1.37)	0.99	0.96 (0.67, 1.38)	0.84	0.88 (0.60, 1.28)	0.50	0.85 (0.56, 1.29)	0.46	1.21 (0.89, 1.64)	0.23	1.41 (0.94, 2.11)	0.094
Respondent age (per 10 years)	0.78 (0.65, 0.95)	0.011	0.92 (0.73, 1.15)	0.45	0.77 (0.62, 0.97)	0.025	0.84 (0.63, 1.12)	0.23	0.84 (0.65, 1.09)	0.19	0.95 (0.69, 1.31)	0.76
Education (5 vs. 0 years)	2.08 (1.62, 2.67)	< 0.001	1.50 (0.97, 2.32)	0.006	2.14 (1.48, 3.08)	< 0.001	1.59 (1.00, 2.51)	0.048	1.88 (1.33, 2.66)	< 0.001	1.46 (0.86, 2.48)	0.16
Marital status		0.79		0.83		0.13		0.051		0.76		0.73
Married/common law (ref)	1		1		1		1		1		1	
Divorced or separated	1.29 (0.47, 3.52)		1.04 (0.35, 3.09)		4.09 (0.67, 25.08)		4.63 (0.99, 21.59)		0.64 (0.07, 6.19)		0.38 (0.06, 2.31)	
Single	0.95 (0.67, 1.34)		0.96 (0.65, 1.41)		0.88 (0.57, 1.36)		0.89 (0.54, 1.46)		0.80 (0.52, 1.24)		1.05 (0.61, 1.79)	
Widowed	0.71 (0.31, 1.64)		0.66 (0.27, 1.61)		0.29 (0.07, 1.17)		0.23 (0.05, 1.00)		1.15 (0.27, 4.89)		1.43 (0.19, 11.09)	
Understands Portuguese	2.01 (1.48, 2.72)	< 0.001	1.34 (0.91, 1.98)	0.14	1.63 (1.08, 2.47)	0.02	0.88 (0.54, 1.46)	0.63	1.52 (1.05, 2.21)	0.028	0.64 (0.36, 1.14)	0.13
Household size (per two members)	0.82 (0.69, 0.97)	0.018	0.81 (0.67, 0.98)	0.031	0.85 (0.68, 1.07)	0.18	0.84 (0.63, 1.12)	0.24	1.03 (0.81, 1.30)	0.81	1.09 (0.81, 1.45)	0.57
Transport to health facility		0.44		0.50		0.12		0.29		0.91		0.69
Bike/motorbike/car (ref)	1		1		1		1		1		1	
On foot	1.44 (0.81, 1.61)		1.13 (0.79, 1.62)		1.41 (0.91, 2.19)		1.30 (0.80, 2.12)		1.02 (0.65, 1.61)		0.90 (0.53, 1.52)	
Travel time to health facility (per 1 hour)	0.80 (0.64, 1.00)	0.047	0.82 (0.57, 1.17)	0.27	0.75 (0.66, 0.86)	< 0.001	0.81 (0.69, 0.94)	0.006	0.62 (0.43, 0.88)	0.008	0.64 (0.30, 1.38)	0.26
Rural vs. urban	0.60 (0.33, 1.11)	0.11	0.87 (0.47, 1.60)	0.65	0.66 (0.32, 1.36)	0.26	0.69 (0.30, 1.58)	0.38	0.82 (0.53, 1.27)	0.37	0.98 (0.58, 1.65)	0.94
District		0.87		0.57				0.69		0.027		0.19
Alto Molócuè (ref)	1		1		1	0.49	1		1		1	
Morrumbala	0.90 (0.54, 1.50)		1.44 (0.80, 2.60)		0.60 (0.29, 1.24)		0.98 (0.48, 1.99)		0.51 (0.28, 0.94)		0.66 (0.32, 1.35)	
Namacurra	1.14 (0.67, 1.94)		1.33 (0.76, 2.34)		0.79 (0.37, 1.71)		1.24 (0.55, 2.80)		0.66 (0.37, 1.15)		0.67 (0.32, 1.38)	
Others	1.03 (0.66, 1.59)		1.34 (0.82, 2.16)		0.97 (0.55, 1.70)		1.35 (0.79, 2.30)		0.54 (0.34, 0.86)		0.52 (0.28, 0.95)	
House has radio	1.60 (1.19, 2.15)	0.002	1.42 (0.98, 2.05)	0.060	1.64 (1.11, 2.41)	0.013	1.44 (0.99, 2.10)	0.058	2.10 (1.40, 3.13)	< 0.001	1.78 (1.17, 2.72)	0.007
Monthly income < 1,000 MZN	0.79 (0.53, 1.19)	0.26	0.86 (0.58, 1.27)	0.45	0.45 (0.25, 0.82)	0.008	0.50 (0.30, 0.85)	0.009	0.45 (0.27, 0.74)	0.002	0.75 (0.45, 1.25)	0.27
Delivered last child in a HCF	3.17 (2.16, 4.67)	< 0.001	2.69 (1.64, 4.40)	< 0.001	2.50 (1.59, 3.92)	< 0.001	1.93 (1.10, 3.37)	0.021	4.16 (2.44, 7.09)	< 0.001	4.43 (2.18, 9.01)	< 0.001
Decision-making: child health care				0.55				0.80		0.003		0.16
Men (ref)	1	0.65	1		1	0.29	1		1		1	
Women	1.01 (0.60, 1.70)		0.91 (0.52, 1.62)		1.32 (0.71, 2.46)		1.08 (0.58, 1.99)		2.13 (1.08, 4.21)		2.01 (0.96, 4.21)	
Both	1.17 (0.72, 1.92)		0.76 (0.41, 1.40)		1.54 (0.89, 2.65)		0.90 (0.43, 1.86)		2.96 (1.58, 5.58)		1.97 (0.89, 4.33)	

CI = confidence interval; HCF = health-care facility.

1. There were 984 children included in the fever model. Missing data were multiply imputed for respondent age (6%), education (< 1%), Portuguese understanding (< 1%), transport (2%), travel time (28%), radio ownership (< 1%), monthly income (23%), delivery in health facility (36%), and decision-making (1%). Modeling without imputation would have resulted in 63% (620/984) casewise deletion.

2. There were 512 children included in the diarrhea model. Missing data were multiply imputed for respondent age (5%), Portuguese understanding (< 1%), transport (2%), travel time (31%), radio ownership (< 1%), monthly income (19%), delivery in health facility (28%), and decision-making (2%). Modeling without imputation would have resulted in 55% (283/512) casewise deletion.

3. There were 543 children included in respiratory illness model. Missing data were multiply imputed for respondent age (6%), Portuguese understanding (< 1%), transport (1%), travel time (31%), radio ownership (< 1%), monthly income (23%), delivery in health facility (29%), and decision-making (1%). Modeling without imputation would have resulted in 58% (325/556) casewise deletion.

a. In the adjusted model, missing values of covariates were accounted for using multiple imputation.

b. Because there was evidence (*P* < 0.10) that the relationship with log-odds of health facility utilization for fever is nonlinear, respondent education is fit using restricted cubic splines.

c. Because there was evidence (*P* < 0.10) that the relationship with log-odds of health facility utilization for respiratory illness is nonlinear, child age is fit using restricted cubic splines.
